# Antivivisection Statistics

**Published:** 1904-10-01

**Authors:** 


### Antivivisection Statistics.

Last week we found it necessary to point out that a letter from Mr. Stephen Coleridge, which had appeared in the daily press, was calculated to mislead the charitable public. Mr. Coleridge, in the letter referred to, declared that the mortality in the London hospitals with medical schools was greater than that at the other general hospitals ; the inference which the public were desired to make being that the great hospitals were unworthy of support. We pointed out that the comparison was not a fair one, and that any deduction from it was unwarrantable, for he had included among his so-called general hospitals without medical schools a number of cottage hospitals and other institutions which do not treat the same proportion of severe and acute cases as find their way to the great hospitals, but had omitted the Poplar Hospital from his list. But whilst discounting the Poplar Hospital, Mr. Coleridge is not so consistently fastidious when he compares the death-rate of the great hospitals with that of such institutions as the Poplar Hospital, because it was devoted entirely to accidents.

This is contrary to fact, as a glance at the annual reports of that Institution show. Further, his plea that he dealt only with general hospitals is equally unacceptable. For example, Mr. Coleridge includes among his "general hospitals" such institutions as the Invalid Asylum, Stoke Newington, and the Hospital of St. John and St. Elizabeth for Incurables, both of which especially point out in their reports that they are not to be considered as general hospitals. The former is designed to help "respectable women and girls above 12, especially servants, when ill, or merely needing rest and change (slight eczema is sometimes admitted)" ; while the latter was "established for the reception of patients suffering from incurable or long-standing disease." It is manifest, therefore, without any labouring of the point, that Mr. Coleridge's figures will not bear scrutiny. Transparent as his methods are to the initiated, it is just possible that they may deceive some few of the benevolent who are strangers to anti-vivisection methods, and it is only for this reason that we have thought it worth while to comment on Mr. Stephen Coleridge's mental gymnastics.

